# Association among oral health, social participation, and higher-level functional capacity in Japanese older people

**DOI:** 10.3389/fdmed.2026.1782479

**Published:** 2026-03-09

**Authors:** Tian Zhu, Nanami Sawada, Michiko Furuta, Toshihide Kimura, Misa Maruoka, Shino Suma, Haruhiko Kashiwazaki, Toru Takeshita

**Affiliations:** 1 Section of Preventive and Public Health Dentistry, Division of Oral Health, Growth and Development, Faculty of Dental Science, Kyushu University, Fukuoka, Japan; 2 Section of Geriatric Dentistry and Perioperative Medicine in Dentistry, Division of Maxillofacial Diagnostic and Surgical Science, Faculty of Dental Science, Kyushu University, Fukuoka, Japan; 3 Manno-Cho Kokumin Kenko Hoken Soda Dental Clinic, Nakatado, Japan; 4 Department of Preventive Dentistry, Graduate School of Biomedical Sciences, Tokushima University, Tokushima, Japan

**Keywords:** higher-level functional capacity, older people, oral health, social participation, teeth

## Abstract

**Background:**

Maintaining a high level of functional ability, which is essential for independent living in older adults, is influenced by social participation. Oral health has been associated with higher-level functional ability; however, whether social participation is related to this association remains unclear. This study aimed to investigate the association among oral health, higher-level functional ability, and social participation.

**Methods:**

This cross-sectional study included 154 participants aged ≥65 years (mean age: 82.6 ± 5.5 years) in an underpopulated area. Higher-level functional ability was assessed using the Tokyo Metropolitan Institute of Gerontology Index of Competence. Oral health was evaluated based on the number of teeth present, occlusal status, and swallowing function. Social participation was measured by the number of participants in social groups, such as sports groups, neighborhood groups, senior citizen clubs, hobby groups, learning and culture clubs, and other types of groups.

**Results:**

After adjusting for age, sex, nutritional status, comorbidities, drug use, cognitive decline, mental health status, marital status, educational level, and going outside, Poisson analysis showed that the number of teeth present was positively associated with higher-level functional ability (B = 0.006, *P* = 0.026). Inclusion of social participation as a covariate attenuated this association (B = 0.005, *P* = 0.089). Occupational status and swallowing function were not associated with higher-level functional ability.

**Conclusion:**

These findings suggest that the number of teeth present is associated with higher-level functional ability and that social participation might affect this association.

## 1 Introduction

With economic development, the increased average life expectancy in Japan, and a significantly declining birthrate, the percentage of older people in Japan is increasing (1). In 2023, the proportion of old people increased to 29.1% (2), leading to a substantial increase in societal expenditures related to care for older adults. Consequently, enhancing the physical and mental health of older adults is necessary to improve their quality of life. It is essential to not only extend the life expectancy but also increase the healthy life expectancy, which refers to the average number of years individuals can live independently (3). The healthy life expectancy in 2016 was 74.79 years for females and 72.14 years for males (4). Thus, improving the healthy life expectancy is a critical component of the aging policy in Japan.

As aging progresses, maintaining a high level of functional capacity is fundamental for independent living in later life. This capacity includes complex daily activities such as shopping, meal preparation, and medication management (5) and represents the ability of older people to manage the more intricate aspects of daily life. Therefore, the maintenance of a high level of functional capacity is crucial for extending healthy life expectancy.

A previous Japanese study found that participation in specific types of social groups reduces the risk of functional disability (6), highlighting the important role of social participation in maintaining functional capacity among older adults. Oral health can contribute to a higher functional capacity and social participation. A cross-sectional study reported that individuals with poor oral health tend to have lower functional capacity scores (7), and similar findings have been confirmed in a longitudinal study (8). In addition, older people who have more teeth participate in more social activities (9). These findings suggest an interrelationship between oral health, functional capacity, and social participation; however, these three factors have not been examined simultaneously in previous studies.

The purpose of this study was to investigate the association between oral health and higher-level functional capacity among older adults and to examine whether social participation affects this association. We hypothesized that poor oral health is associated with a lower functional capacity and that social participation may influence this association.

## 2 Methods

### 2.1 Study population

This cross-sectional study was conducted from 2017 to 2018 in Kotonami, Manno Town, Nakatado District, Kagawa Prefecture, Japan (10). The district was classified as a depopulated area, and 48.2% of the total population of 2,183 was aged ≥65 years. Participants were included if they fulfilled the following criteria: (i) community-dwelling individuals aged ≥65 years, (ii) those who lived at home, (iii) those who did not reside in nursing care facilities, and (iv) those who attended dental examinations and questionnaire surveys. Dental examinations and questionnaire surveys were conducted when the examiners visited the home or when the individuals received preventive day care services under Japan's long-term care insurance system. In total of 252 participants (aged 65–74 years [*n* = 7] and those aged ≥75 years [*n* = 245]) who met the inclusion criteria were identified. The participation rate of residents aged ≥75 years was 46.8%. Participants were excluded if they had incomplete data on oral health, social participation, or a higher level of functional capacity.

The required sample size was calculated using G*Power software. The calculation was based on an effect size of 0.52, as estimated in a previous study (7), a statistical power of 80%, and a significance level of 5%. The required sample size was estimated to be 120 participants. However, this study exceeded this threshold, ensuring sufficient power to detect an association between oral health and higher-level functional capacity.

This study was approved by the Kyushu University Institutional Review Board for Clinical Research (approval no. 24363). Written informed consent was obtained from all participants.

### 2.2 Oral condition

Three dimensions were used to assess oral health: the number of teeth present, occlusal status, and swallowing function. The number of teeth present was defined as the number of naturally existing teeth, excluding the third molars, dentures, and retained roots. The maximum number of teeth present was 28. The occlusal status was categorized into two groups: none/unilateral and bilateral occlusion. Swallowing function was evaluated using the Modified Water Swallowing Test (MWST), a validated screening tool in which participants were asked to swallow a small amount of water to identify signs of swallowing difficulty, such as choking (11). The MWST was scored on a scale of 1–5. In this study, a score of ≤3 indicated suspected swallowing dysfunction, while a score >3 indicated normal swallowing function (11, 12).

### 2.3 Higher-level functional capacity

Higher-level functional capacity was assessed using the Tokyo Metropolitan Institute of Gerontology Index of Competence (TMIG-IC), a multidimensional 13-item scale (13). This index corresponds to the fifth, sixth, and seventh sublevels of Lawton's model of competence (14) and is designed to evaluate advanced activities of daily living. The maximum total TMIG-IC score was 13, with higher scores indicating greater functional competence. TMIG-IC comprised three subscales: instrumental self-maintenance (IADL), intellectual activity, and social role (13). The IADL subscale (items 1–5) assessed practical daily tasks, such as using public transportation independently and managing finances. The intellectual activity subscale (items 6–9) evaluated activities related to cognitive engagement, such as reading books or newspapers. The social role subscale (items 10–13) measured aspects of social interaction, such as visiting friends, providing advice to others, visiting ill friends, and communicating with younger individuals.

### 2.4 Social participation

Social participation was evaluated by counting the number of social groups attended by the participants. Based on modifications of the method of Takeuchi et al. (9), social groups included sports clubs, neighborhood associations, senior community clubs, hobby groups, learning activities, and other types of organizations. The maximum score for social participation was six, with one point for participation in each category.

### 2.5 Physical and mental health conditions

Other health-related measures included cognitive decline, mental health, comorbidities, and nutritional status. Cognitive decline was assessed using the Mini-Cog, a screening tool consisting of two components: a clock-drawing task based on a specified time and a three-word recall test (15). The total score ranged from 0 to 5, and scores of ≤2 indicated suspected cognitive impairment. Mental health was evaluated using the six-item Kessler Psychological Distress Scale (K6), which had a maximum score of 24; higher scores reflected greater psychological distress and a higher likelihood of depression (16). Comorbidities were assessed using the Charlson Comorbidity Index (CCI); the disease severities were weighted from 1 to 6 points, and the total CCI score represented the overall comorbidity burden (17). The nutritional status was assessed using the Mini Nutritional Assessment Short-Form (MNA-SF), which consisted of six items evaluating weight loss, appetite, body mass index (BMI), and psychological stress (18). The maximum score was 14, and a score of ≤11 indicated a risk of malnutrition.

### 2.6 Other measures

Medication use was categorized into two groups: individuals taking ≥3 types of medication daily and those taking <3 types. The marital status was classified into two groups: married and widowed or divorced. The educational level was categorized into three groups: 0–9, 10–12, and ≥13 years. The frequency of going outside was divided into two groups: those who went outside daily and those who did not.

### 2.7 Statistical analysis

Descriptive statistics were employed to assess the oral condition, higher-level functional capacity, and physical and mental conditions, with continuous values presented as median (interquartile range) and categorical values expressed as numbers and percentages. Participants with missing data on oral health, social participation, higher-level functional capacity, and other related-factors were excluded from the analysis. The higher-level functional capacity, assessed using the TMIG-IC score, was used as a nonparametric distributed continuous variable. For each subscale of the TMIG-IC, including the IADL, intellectual activity, and social roles, participants who achieved full scores were classified as normal in that domain, whereas those who scored less than full scores were considered impaired.

The Mann–Whitney U and Kruskal–Wallis tests were used to examine the associations between categorical variables and TMIG-IC scores. Spearman's rank correlation analysis was used to assess the association between continuous variables and TMIG-IC scores. To identify the association between the number of teeth present and TMIG-IC score, multivariate analyses were conducted using the Poisson regression model. Additionally, binary logistic regression analysis was performed to examine the association between the number of teeth present and TMIG-IC subscales, including the IADL, intellectual activity, and social roles. Three different models were developed to determine whether the association between the number of teeth present and TMIG-IC score was influenced by the inclusion of going outside and social participation. Model 1 included the number of teeth present as the independent variable, the TMIG-IC score as the dependent variable, and age, sex, occlusal status, swallowing function, nutritional status, comorbidities, use of medicines, cognitive decline, mental health, marital status, and educational level as covariates. Model 2 included the covariates in Model 1 with the addition of the frequency of going outside (19), which has been associated with oral health and higher-level functional capacity (20). Model 3 included the covariates in Model 2 and the addition of the number of social participation activities. All statistical analyses were conducted using SPSS software (version 29.0; IBM Corp., Armonk, NY, USA), and *p*-values < 0.05 were considered statistically significant.

## 3 Results

Of the 252 participants who completed the survey, 98 were excluded because of missing data. The final study population consisted of 154 participants (mean age: 82.6 years; 40 men, 114 women). The participant characteristics are listed in Table 1. The mean participant age was 82.6 years [standard deviation (SD) = 5.5], and the mean TMIG-IC score was 10.0 (SD = 3.3). The average number of social participation activities was 1.5 (SD = 1.5).

**Table 1.** Characteristics of the participants

Variables	Mean ± SD / n (%)
Age (y)	82.6 ± 5.5
Sex	
Male	40 (26.0)
Female	114 (74.0)
Number of teeth present	13.1 ± 10.4
Denture use	
No	48 (31.2)
Yes	106 (68.8)
Occlusal contact of molars	
None/unilateral	19 (12.3)
Bilateral	135 (87.7)
Swallowing function (MWST)	
Suspected decline	7 (4.5)
Non-suspected decline	147 (95.5)
Nutritional status (MNA-SF)	
Malnutrition/at risk of malnutrition	43 (27.9)
Well-nourished	111 (72.1)
Higher-level functional capacity (TMIG-IC score)	10.0 ± 3.3
Dependent in IADL	59 (38.3)
Dependent in IA	76 (49.4)
Dependent in SR	81 (52.6)
Comorbidities (CCI)	0.96 ± 1.5
Number of medications	
≥3	113 (73.4)
<3	41 (26.6)
Cognitive function	
Suspected decline	28 (18.2)
Non-suspected decline	126 (81.8)
Mental health status (K6)	4.4 ± 4.4
Number of social participation activities	1.5 ± 1.5
Frequency of going out	
Not almost every day	120 (77.9)
Almost every day	34 (22.1)
Marital status	
Unmarried	0 (0.0)
Widowed/divorced	79 (51.3)
Married	75 (48.7)
Educational level	
0–9 years	74 (48.1)
10–12 years	54 (35.1)
≥13 years	26 (16.9)

SD, standard deviation; MWST, Modified Water Swallowing Test; MNA-SF, Mini Nutritional Assessment-Short Form; TMIG-IC, Tokyo Metropolitan Institute of Gerontology Index of Competence; IADL, instrumental activities of daily living; IA, intellectual activity; SR, social role; CCI, Charlson Comorbidity Index; K6, 6-item Kessler Psychological Distress Scale.

Tables 2, 3 present the results of the univariate analysis of the association between the TMIC-IC score and related factors. Regarding the oral condition, the occlusal condition and swallowing function were not associated with the TMIG-IC score (Table 2), while the number of teeth present was positively associated with the TMIG-IC score (Table 3). The number of social participation activities was positively correlated with the TMIG-IC score (Table 3). Taking ≥3 types of medications daily, suspected cognitive decline, not going outside daily, and being widowed or divorced were associated with lower TMIG-IC scores (Table 2). Age and comorbidities were negatively associated with the TMIG-IC score (Table 3).

**Table 2:** Association between categorical variables and higher-level functional capacity (TMIG-IC score).

Variables	TMIG-IC score Median (25th percentile, 75th percentile).	*P*-value
Sex		
Male	11 (9, 13)	0.383
Female	11 (8, 12)	
Occlusal contact of molars		
None/Unilateral	11 (6, 13)	0.967
Bilateral	11 (9, 12)	
Swallowing function (MWST)		
Suspected decline	10 (10, 12)	0.444
Non-suspected	11 (8, 13)	
Nutritional status (MNA-SF)		
Malnutrition/at risk of malnutrition	11 (7, 12)	0.097
Well-nourished	11 (9, 13)	
Number of medicatons		
≥3	11 (7, 12)	<0.001
<3	12 (11, 13)	
Cognitive function		
Suspected decline	7 (2.5, 9.75)	<0.001
Non-suspected	12 (10, 13)	
Frequency of going out		
Not almost every day	11 (8, 12)	0.008
Almost every day	12 (10, 13)	
Marital status		
Widowed/divorced	12 (10, 13)	<0.001
Married	10 (7, 12)	
Educational level		
0–9 years	11 (8, 12)	0.100^a^
10–12 years	11 (8, 12)	
≥13 years	11 (10, 13)	

Mann–Whitney *U*-test.

^a^ Kruskal–Wallis test.

TMIG-IC, Tokyo Metropolitan Institute of Gerontology Index of Competence; IQR, interquartile range; MWST, Modified Water Swallowing Test; MNA-SF, Mini Nutritional Assessment-Short Form.

**Table 3:** Association between continuous variables and higher-level functional capacity (TMIG-IC score).

Variables	r	*P*-value
Age	−0.388	<0.001
Number of teeth present	0.413	<0.001
Comorbidities (CCI)	−0.306	<0.001
Mental health status (K6)	−0.132	0.104
Number of social participation activities	0.509	<0.001

Spearman's rank correlation coefficient.

TMIG-IC, Tokyo Metropolitan Institute of Gerontology Index of Competence; CCI, Charlson Comorbidity Index; K6, 6-item Kessler Psychological Distress Scale.

Table 4 summarizes the results of the three Poisson regression models examining whether the association between the number of teeth present and the TMIG-IC score was influenced by additional factors. In Model 1, which was adjusted for age, sex, occlusal status, swallowing function, nutritional status, comorbidities, medication use, suspected cognitive decline, mental health, marital status, and educational level, the number of teeth present was positively associated with the TMIG-IC score (B = 0.006, 95% CI: 0.001–0.012, *P* = 0.025). In Model 2, which included the frequency of going outside with the covariates in Model 1, the positive association remained significant (B = 0.006, 95% CI: 0.001–0.012, *P* = 0.026). In Model 3, which further included the number of social participation activities, the association between the number of teeth present and the TMIG-IC score was attenuated and no longer statistically significant (B = 0.005, 95% CI: −0.001–0.011, *P* = 0.089).

**Table 4.** Poisson regression analysis with higher-level functional capacity (TMIG-IC score) as the dependent variable

Variables	**Model 1**			**Model 2**			**Model 3**		
	B	95%CI	P-value	B	95%CI	P-value	B	95%CI	P-value
Number of teeth present	0.006	(0.001, 0.012)	0.025	0.006	(0.001, 0.012)	0.026	0.005	(-0.001, 0.011)	0.089
Age	-0.008	(-0.020, 0.004)	0.209	-0.008	(-0.020, 0.004)	0.211	-0.005	(-0.017, 0.007)	0.409
Sex (ref. female)									
Male	-0.022	(-0.151, 0.108)	0.745	-0.024	(-0.154, 0.106)	0.716	-0.013	(-0.143, 0.117)	0.841
Occlusal contact of molars (ref. bilateral)									
None/unilateral	0.032	(-0.132, 0.195)	0.702	0.028	(-0.136, 0.191)	0.738	0.038	(-0.126, 0.201)	0.651
Swallowing function (MWST) (ref. non-suspected)									
Suspected decline	-0.087	(-0.349, 0.174)	0.512	-0.087	(-0.348, 0.175)	0.515	-0.055	(-0.318, 0.208)	0.683
Nutritional status (MNA-SF) (ref. well-nourished)									
Malnutrition/at risk of malnutrition	-0.044	(-0.161, 0.073)	0.458	-0.036	(-0.155, 0.082)	0.550	-0.050	(-0.169, 0.069)	0.412
Comorbidities (CCI)	0.003	(-0.038, 0.044)	0.871	0.005	(-0.036, 0.046)	0.811	0.009	(-0.032, 0.050)	0.669
Number of medications (ref. <3)									
≥3	-0.108	(-0.230, 0.013)	0.079	-0.100	(-0.222, 0.023)	0.112	-0.091	(-0.214, 0.032)	0.146
Cognitive function (ref. non-suspected)									
Suspected decline	-0.404	(-0.572, -0.236)	<0.001	-0.399	(-0.567, -0.230)	<0.001	-0.382	(-0.551, -0.212)	<0.001
Mental health status (K6)	-0.010	(-0.023, 0.002)	0.102	-0.010	(-0.022, 0.003)	0.131	-0.006	(-0.019, 0.006)	0.326
Marital status (ref. married)									
Bereavement/divorce	-0.029	(-0.151, 0.094)	0.646	-0.025	(-0.148, 0.098)	0.688	-0.040	(-0.163, 0.082)	0.518
Educational level (ref. ≥13 years)									
0–9 years	0.022	(-0.128, 0.172)	0.777	0.003	(-0.152, 0.159)	0.968	0.050	(-0.110, 0.210)	0.542
10–12 years	-0.042	(-0.19, 0.107)	0.580	-0.046	(-0.196, 0.103)	0.541	0.002	(-0.152, 0.156)	0.979
Frequency of going out (ref. almost every day)									
Not almost every day				-0.059	(-0.187, 0.069)	0.368	-0.031	(-0.161, 0.099)	0.641
Number of social participation activities							0.048	(0.011, 0.084)	0.010

Independent variables

Model 1: Number of teeth present, age, sex, occlusal contact of molars, swallowing function, nutritional status, comorbidities, number of medications, cognitive function, mental health status, marital status, and educational level.

Model 2: Frequency of going out was added to Model 1.

Model 3: Number of participating communities was added to Model 2.

TMIG-IC, Tokyo Metropolitan Institute of Gerontology Index of Competence; CI, confidence interval; MWST, Modified Water Swallowing Test; MNA-SF, Mini Nutritional Assessment-Short Form; CCI, Charlson Comorbidity Index; K6, 6-item Kessler Psychological Distress Scale.

When we investigated the association between denture wearing and the TMIG-IC score, denture wearing was not associated with the TMIG-IC score (B = −0.071, 95% CI: −0.225–0.082, *P* = 0.362) in Model 3. The association between the number of teeth present and TMIC-IC score was still attenuated (B = 0.007, 95% CI: 0.001–0.015, *P* = 0.060), while a significant association with social participation activities was found.

Table 5 shows the results of the binary logistic regression analysis with the TMIC-IC subscales, such as the IADL, intellectual activity, and social roles, as the dependent variables. Participants who achieved full scores were classified as normal in that domain, whereas those who scored less than full scores were considered impaired. The number of teeth present was associated with normal intellectual activity in Model 3, which included all covariates. However, no association between the number of teeth and normal IADL or social roles was observed.

**Table 5:** Logistic regression analysis with subscales of higher-level functional capacity (TMIG-IC) as the dependent variable.

Variables	Model 1			Model 2			Model 3		
	OR	95%CI	*P*-value	OR	95%CI	*P*-value	OR	95%CI	*P*-value
Normal IADL									
Number of teeth present	1.030	(0.985, 1.077)	0.199	1.030	(0.984, 1.078)	0.205	1.017	(0.967, 1.070)	0.508
Frequency of going out				2.654	(0.723, 9.740)	0.141	1.890	(0.478, 7.476)	0.364
Number of social participation activities							2.236	(1.399, 3.575)	<0.001
Normal intellectual activity									
Number of teeth present	1.059	(1.018, 1.103)	0.005	1.060	(1.018, 1.103)	0.005	1.056	(1.014, 1.100)	0.008
Frequency of going out				0.730	(0.280, 1.904)	0.520	0.662	(0.248, 1.770)	0.411
Number of social participation activities							1.206	(0.913, 1.593)	0.188
Normal social roles									
Number of teeth present	1.003	(0.966, 1.043)	0.863	1.004	(0.965, 1.044)	0.843	0.996	(0.956, 1.037)	0.841
Frequency of going out				3.648	(1.377, 9.667)	0.009	3.088	(1.147, 8.313)	0.026
Number of social participation activities							1.346	(1.011, 1.793)	0.042

Independent variables.

Model 1: Number of teeth present, age, sex, occlusal contact of molars, swallowing function, nutritional status, comorbidities, number of medications, cognitive function, mental health status, marital status, and educational level.

Model 2: Frequency of going out was added to Model 1.

Model 3: Number of participating communities was added to Model 2.

TMIG-IC, Tokyo Metropolitan Institute of Gerontology Index of Competence; IADL, instrumental activities of daily living; OR, odds ratio; CI, confidence interval.

## 4 Discussion

In this study, the number of teeth present was associated with higher-level functional capacity, as measured by the TMIG-IC score, even after adjusting for covariates. Similarly, the extent of social participation (i.e., the number of social participation groups) was associated with the TMIG-IC score. These findings align with those of previous studies indicating that having more teeth (7) or greater social participation (21) is associated with better IADL performance. Notably, the association between the number of teeth present and TMIG-IC score was attenuated when social participation was included in the model, suggesting a potential mediating role. To the best of our knowledge, this is the first study to examine whether social participation affects the association between dentition and higher-level functional capacity.

A plausible pathway connecting the number of teeth with higher-level functional capacity is through social participation, which is influenced by both the functional and esthetic aspects of dentition. Teeth support key social behaviors, including smiling, clear speech, and eating and help maintain facial appearance (22). Therefore, tooth loss can undermine confidence and reduce social engagement, whereas retaining more teeth may facilitate participation (23). Social participation fosters interactions within the community and, among older adults, is associated with a greater sense of fulfillment, reduced social withdrawal and loneliness, improved exposure to health-promotion information, and an enhanced ability to buffer stress-related adverse effects on mental and physical health (24). Collectively, these processes may help preserve higher-level functional capacity and slow its decline.

The association between the number of teeth present and higher-level functional capacity was not influenced by the frequency of going out. Although going out, particularly among retirees, is often emphasized as a way to reduce social isolation and promote both physical activity and social contact (25), the frequency of going out may index physical activity more than social engagement. When physical disability was assessed by care needs level in the long-term care insurance system in Japan (26), care needs level was negatively correlated with social participation activities in this study (Spearman correlation coefficient = −0.503, *P* < 0.001). However, the association between the number of teeth present and higher-level functional capacity (B = 0.006, 95% CI: 0.001–0.012, *P* = 0.031) was not influenced by care needs level. Accordingly, social engagement rather than physical activity or physical disability may be a more relevant pathway linking dentition to higher-level functional capacity.

When the TMIG-IC was divided into three subscales–IADL, intellectual activity, and social role–only intellectual activity was significantly associated with the number of teeth present. This association remained independent of cognitive function or educational level. One possible explanation is health literacy, which may explain the association between the number of teeth present and intellectual activity. Previous studies have reported that health literacy is more strongly related to the intellectual activity subscale of the TMIG-IC than to the other subscales (27). Health literacy has also been closely linked with good oral health (28). The observed association between the number of teeth present and intellectual activity may be partly explained by the underlying health literacy.

Social participation affected the association between the number of teeth present and overall TMIG-IC score, suggesting that having more teeth may facilitate higher-level functional capacity, partly through increased opportunities for social engagement. However, social participation did not affect the association between the number of teeth present and the intellectual activity subscale. This may be attributable to the fact that intellectual activities, such as reading newspapers and books, depend primarily on individual resources that are less associated with social interactions. Therefore, the effects of social participation do not extend to intellectual activity.

This study had several limitations. First, because this was a cross-sectional study, we could not determine the temporal relationship between the number of teeth present, social participation, and higher-level functional capacity, because these factors were measured simultaneously. Nonetheless, attenuation of the association between the number of teeth present and the TMIG-IC score after adjusting for social participation suggests that the number of teeth present is unlikely to be directly related to the TMIG-IC score. Social participation may act as a mediator in this association. However, we did not employ causal inference methods such as mediation analysis because the causal pathways among these factors could not be established in the present study. Future studies are needed to investigate these longitudinal associations. Second, health literacy was not assessed in this study and may have influenced the association between the number of teeth present and higher-level functional capacity. Third, this study did not include individuals residing in nursing care facilities or those with missing data. This may have contributed to the relatively higher TMIG-IC scores (43.5% of the participants scored 12 or 13) in the study population, which may have induced a ceiling effect. This ceiling effect may have attenuated the association between the number of teeth present and higher-level functional capacity. Finally, this study was conducted in rural areas, where demographic characteristics, access to dental care, and social groups may differ from those in urban settings. Therefore, caution should be exercised when generalizing these findings to other populations.

In conclusion, this study found that the number of teeth present was positively associated with higher-level functional capacity and that this association was influenced by social participation. Future longitudinal studies are required to clarify the causal nature of this relationship.

## Data Availability

The datasets presented in this article are not readily available due to ethical restriction. Requests to access the datasets should be directed to Michiko Furuta, mfuruta@dent.kyushu-u.ac.jp.
